# Stump Appendicitis Seven Years after Appendectomy

**Published:** 2013-07-31

**Authors:** S Crocco, F Pederiva, E Zanelli, M Scarpa, E Barbi, A Ventura

**Affiliations:** Pediatric Emergency Department, Institute for Maternal and Child Health - IRCCS “Burlo Garofolo” – Trieste, Italy; Pediatric Surgery, Institute for Maternal and Child Health - IRCCS “Burlo Garofolo” – Trieste, Italy; Pediatric Emergency Department, Institute for Maternal and Child Health - IRCCS “Burlo Garofolo” – Trieste, Italy; Pediatric Surgery, Institute for Maternal and Child Health - IRCCS “Burlo Garofolo” – Trieste, Italy; Pediatric Emergency Department, Institute for Maternal and Child Health - IRCCS “Burlo Garofolo” – Trieste, Italy; Pediatric Clinical Department, Institute for Maternal and Child Health - IRCCS “Burlo Garofolo” – Trieste, Italy

A 15-year-old boy was admitted to our hospital with abdominal pain and a surgical history of video-assisted transumbilical appendectomy for a gangrenous appendicitis 7 years back. The small stump was closed without purse-string suture. The postoperative period was complicated by an intraperitoneal abscess treated with antibiotics. Later on he experienced multiple episodes (thrice a year) of self limiting intermittent abdominal pain with pallor, sweating and diarrhea. During current admission, the abdominal pain started 3 days back. It was described as colic, getting worse and associated with pallor and sweating. There was no fever, vomiting, diarrhea, or urinary symptoms. Vital signs were normal. Physical examination revealed a subumbilical scar and peritoneal irritation in the right lower quadrant; no palpable masses were appreciated and genitourinary examination was unremarkable. The abdomen was not distended but bowel sounds were decreased. Laboratory data showed an increased white blood cell count of 10,570 cells/mm with 74.2% neutrophils, CRP 5.41 mg/dl and ESR 4 mm/h. Electrolytes, urinalysis, transaminases and amylase were all in the normal range. Abdominal ultrasound demonstrated ileocecal wall thickening (3-6 mm) with increased signals in the vascular color-doppler and hyperplasia of the surrounding fatty tissue. A small amount of corpuscular pericecal free fluid extending to Douglas pouch was noticed (Fig. 1, 2). CT scan showed a mixed cystic and solid mass (6.5 cm x 6 cm x 4.2 cm) in the right lower quadrant with a calcified fecolith (Fig. 3). The presumptive preoperative diagnosis was stump appendicitis. At operation, a 5-cm-long appendiceal stump with a perforation at the base and a fecolith outside of the lumen was found and removed. A purse-string suture was placed to close the cecal wall at the base of the removed stump. Histopathology showed acute inflammation and patchy necrosis of the appendiceal stump. The post-operative course was uneventful.

**Figure:**
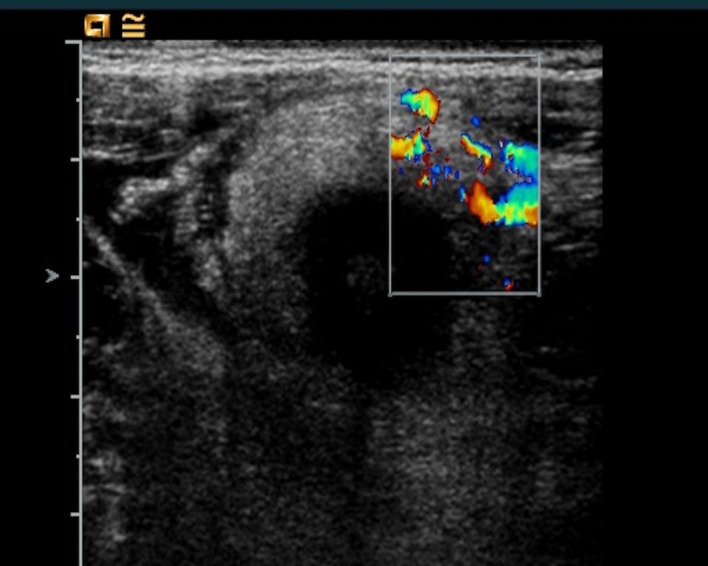
Figure 1:Abdominal ultrasound image of ileocecal wall thickening (3-6 mm) with increased signal in the vascular color-doppler and hyperplasia of the surrounding fatty tissue.

**Figure:**
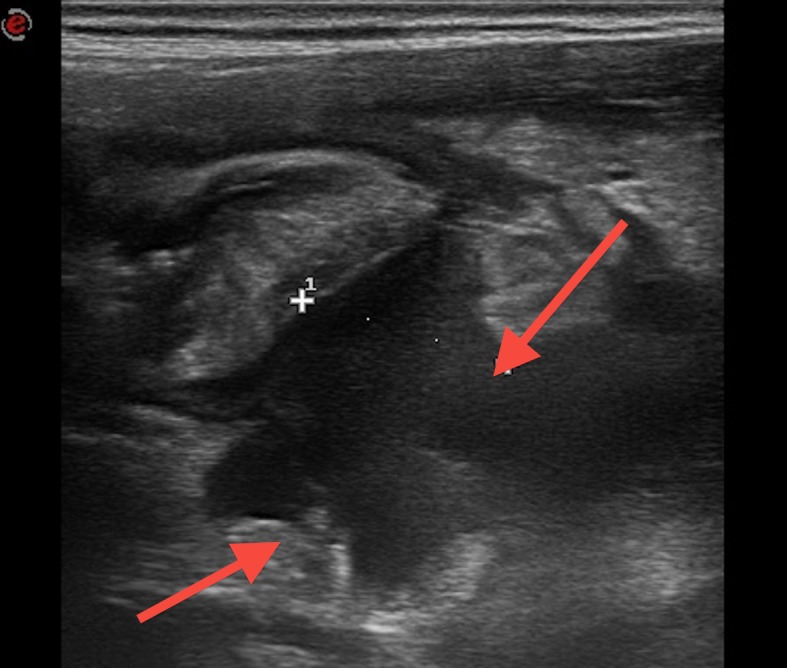
Figure 2:Abdominal ultrasound image showing a small amount of corpuscular pericecal free fluid extending to pouch of Douglas

**Figure:**
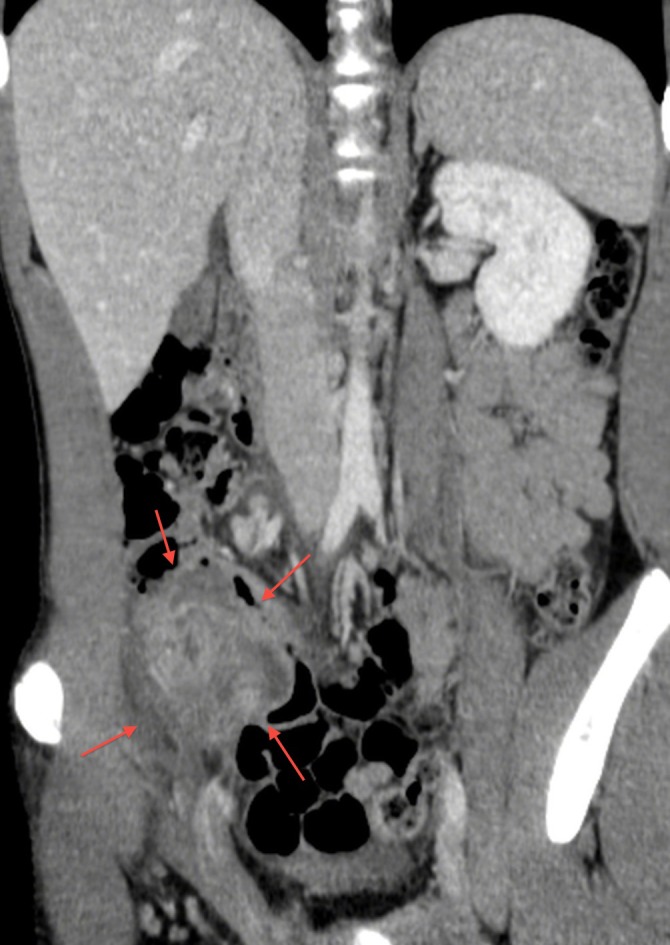
Figure 3:CT image of a mixed cystic and solid mass (6.5 cm x 6 cm x 4.2 cm) in the right lower quadrant with a calcified fecolith.

## DISCUSSION

Stump appendicitis is the re-inflammation of the residual appendiceal tissue after an appendectomy. It represents a rare delayed complication of appendectomy the exact incidence of which is not known. In the cases described in literature the time from appendectomy to the development of stump appendicitis ranges from few weeks to decades.[1,2] Incomplete appendectomy leaving a stump longer than 5 mm, severe local inflammation preventing adequate identification of the appendiceal base, retrocecal or subserosal appendix, and, last but not least, the insufficient experience of the surgeon may influence the occurrence of this condition.[1-5] Moreover some sources have suggested that the growing use of laparoscopic appendectomy may increase the frequency of stump appendicitis. This may be the result of leaving a longer stump, secondary to a smaller field of vision, lack of three-dimensional perspective, and the absence of tactile feedback.[6,7] The incidence of stump appendicitis can be minimized simply by adequately visualizing the base of the appendix and creating a stump less than 3 mm in length. Therefore, if performed properly, there is no reason why laparoscopic appendectomy should lead to a higher incidence of stump appendicitis.

Clinical features of stump appendicitis do not differ from that of acute appendicitis, even though the history of a previous appendectomy can be misleading causing a delay in diagnosis. Ultrasonography can be useful in identifying inflammatory changes. The abdominal CT is reported as the gold standard for the diagnosis and should be considered as the initial diagnostic study in patients with right lower quadrant symptoms after appendectomy. It allows the recognition of retained fecolith or postoperative abscess as well as cases of stump appendicitis.[1] Stump appendicitis has a higher risk of complications with perforation being reported in nearly 70% of the cases. It is therefore imperative that patients with stump appendicitis undergo complete appendectomy as soon as possible. Stump appendicitis should be considered in any patient with a previous history of appendectomy and a convincing history of acute appendicitis. Treatment by re-surgery and complete removal of the appendix will resolve the case.

## Footnotes

**Source of Support:** Nil

**Conflict of Interest:** None declared

